# Efficacy and safety of spesolimab in Asian patients with a generalized pustular psoriasis flare: Results from the randomized, double‐blind, placebo‐controlled Effisayil™ 1 study

**DOI:** 10.1111/1346-8138.16609

**Published:** 2022-10-25

**Authors:** Akimichi Morita, Tsen‐Fang Tsai, Evelyn Yap Wen Yee, Yukari Okubo, Shinichi Imafuku, Min Zheng, Ling Li, Manuel Quaresma, Christian Thoma, Siew Eng Choon

**Affiliations:** ^1^ Department of Geriatric and Environmental Dermatology Nagoya City University, Graduate School of Medical Sciences Nagoya Japan; ^2^ Department of Dermatology National Taiwan University Hospital and National Taiwan University College of Medicine Taipei Taiwan; ^3^ Department of Dermatology Hospital Pakar Sultanah Fatimah Muar Malaysia; ^4^ Department of Dermatology Tokyo Medical University Tokyo Japan; ^5^ Department of Dermatology, Faculty of Medicine Fukuoka University Fukuoka Japan; ^6^ Department of Dermatology, Second Affiliated Hospital School of Medicine, Zhejiang University Hangzhou China; ^7^ Boehringer Ingelheim (China) Investment Corporation Limited Shanghai People's Republic of China; ^8^ Boehringer Ingelheim International GmbH Ingelheim Germany; ^9^ Boehringer Ingelheim International GmbH Biberach Germany; ^10^ Department of Dermatology Hospital Sultanah Aminah, Johor Bahru, Malaysia, and Clinical School Johor Bahru, Monash University Malaysia Johor Bahru Malaysia

**Keywords:** Asians, clinical trial, generalized pustular psoriasis, GPP, psoriasis, spesolimab

## Abstract

Generalized pustular psoriasis is a potentially life‐threatening neutrophilic skin disease characterized by recurrent flares of widespread erythema and eruption of sterile pustules. In the Effisayil™ 1 study (NCT03782792), 53 patients with a generalized pustular psoriasis flare were treated with placebo or spesolimab, a humanized anti‐interleukin‐36 receptor monoclonal antibody, the first targeted treatment to be studied in a randomized clinical trial. Spesolimab treatment resulted in rapid pustular and skin clearance, with an acceptable safety profile. Here, we evaluate the efficacy and safety of spesolimab in 29 Asian patients in the Effisayil™ 1 study. The primary endpoint, a Generalized Pustular Psoriasis Physician Global Assessment (GPPGA) pustulation subscore of 0 (no visible pustules) at Week 1, was achieved by 10 patients (62.5%) randomized to spesolimab and one patient (7.7%) randomized to placebo (risk difference 54.8, 95% confidence interval [CI] 17.3–79.8). The key secondary endpoint, a GPPGA total score of 0 or 1 (clear or almost clear skin) at Week 1, was achieved by eight (50.0%) and two (15.4%) patients, respectively (risk difference 34.6, 95% CI −3.1–64.7). This was similar to previously published data in the overall population in whom the primary and key secondary endpoints were achieved by 54% versus 6% and 43% versus 11% of patients, respectively. The percentages of Asian patients randomized to spesolimab with a GPPGA pustulation subscore of 0 and GPPGA total score of 0 or 1 were sustained above 60% for up to 12 weeks. In these patients, patient‐reported outcomes also improved and markers of systemic inflammation were normalized. Eleven (68.8%) and eight (61.5%) of spesolimab‐ and placebo‐treated patients, respectively, experienced at least one adverse event. In conclusion, spesolimab improved outcomes in Asian patients compared with placebo, supporting its use in the treatment of generalized pustular psoriasis flares.

## 1 INTRODUCTION

Generalized pustular psoriasis (GPP) is a potentially life‐threatening neutrophilic skin disease characterized by recurrent flares of widespread erythema with eruption of sterile pustules that can coalesce to form lakes of pus.^1^, ^2^, ^3^, ^4^, ^5^ GPP is a rare disease that has been suggested to be around five times more prevalent in Asia than in Europe, with a report in Japan estimating 7.46 cases per million persons.^6^, ^7^ However, evidence from electronic databases indicates that the prevalence may in fact be similar in Asia and Europe, with a recent Swedish study reporting 91 cases per million persons.^8^ Compared with other forms of psoriasis, patients with GPP exhibit a lower body mass index (BMI), lower likelihood of being a habitual drinker, and higher probability of being female.^9^

GPP flares may be associated with systemic inflammation and can present with symptoms such as fever, fatigue, and leukocytosis. Consequently, there can be a severe impact on patient quality of life and there are risks of serious complications requiring hospitalization, such as sepsis, acute respiratory distress syndrome, renal failure, and congestive heart failure.^10^, ^11^, ^12^, ^13^, ^14^, ^15^ Historically, the management of GPP has been based largely on strategies optimized for managing plaque psoriasis. However, plaque psoriasis is phenotypically, genetically, and histopathologically distinct from GPP,^16^ necessitating a different treatment strategy.^1^, ^4^, ^17^, ^18^, ^19^, ^20^, ^21^ Currently, globally accepted guidelines for the management of GPP are lacking. Biologics targeting proinflammatory pathways have been approved for use in patients with GPP in some Asian countries, including Japan,^2^, ^22^, ^23^, ^24^ despite limited evidence from open‐label, single‐arm clinical trials with very small sample sizes.^2^, ^25^, ^26^, ^27^, ^28^, ^29^, ^30^, ^31^, ^32^ There is therefore a need for treatments that have been proven to control GPP flares.

Evidence suggests that the interleukin (IL)‐36 pathway plays a key role in the pathology of GPP, and several studies have indicated that Asian patients with GPP have high rates (46.8%–81.8%) of mutations in *IL36RN*, the gene encoding the IL‐36 receptor antagonist. As such, blockade of the IL‐36 pathway could be an effective therapeutic approach for GPP.^1^, ^5^, ^18^, ^33^, ^34^, ^35^, ^36^, ^37^, ^38^

In the Effisayil™ 1 study (NCT03782792), patients with a GPP flare treated with spesolimab, a humanized anti‐IL‐36 receptor monoclonal antibody and the first targeted treatment for GPP to be studied in a randomized clinical trial, achieved rapid pustular and skin clearance with an acceptable safety profile.^39^ At Week 1, the primary endpoint (Generalized Pustular Psoriasis Physician Global Assessment [GPPGA] pustulation subscore of 0, no visible pustules) was achieved by 54% of patients receiving spesolimab compared with 6% receiving placebo (one‐sided *p* < 0.001). At Week 1, the key secondary endpoint (GPPGA total score of 0 or 1, clear or almost clear skin) was achieved by 43% of patients receiving spesolimab compared with 11% receiving placebo (one‐sided *p* = 0.0118). In this analysis, we evaluate the efficacy and safety of spesolimab in Asian patients with a GPP flare in the Effisayil™ 1 study.

## 2 METHODS

### 2.1 Study design and patients

Effisayil™ 1 was a multicenter, randomized, double‐blind, placebo‐controlled study. The methods have been published previously and are therefore described only briefly here.^39^ Patients aged 18–75 years presenting with a GPP flare were randomized (2:1) to receive a single intravenous dose of spesolimab 900 mg or placebo and were followed for 12 weeks. During Week 1, if disease worsened despite spesolimab treatment, other treatments for GPP could be added at the investigator's discretion. Patients in either group with persistent GPP flare symptoms (GPPGA pustulation/total score ≥2) could be given one open‐label spesolimab infusion on Day 8. After Week 1, patients with a clinical response (GPPGA total score 0 or 1) who had a new flare (GPPGA pustulation/total score ≥2) could receive one open‐label spesolimab infusion; patients without a clinical response who had a new flare could receive other treatment for GPP at the investigator's discretion.

### 2.2 Study endpoints

The primary endpoint was a GPPGA pustulation subscore of 0 (no visible pustules) at the end of Week 1. The key secondary endpoint was a GPPGA total score of 0 or 1 (clear or almost clear skin) at the end of Week 1. Patient‐reported outcomes (PROs) were pain visual analog scale (pain VAS), Functional Assessment of Chronic Illness Therapy–Fatigue (FACIT‐Fatigue), Dermatology Quality of Life Index (DLQI), and Psoriasis Symptom Scale (PSS). For these scales, minimal clinically important differences (MCIDs) were defined as a 30‐point decrease, a 4‐point increase, a 4‐point decrease, and a 2‐point decrease, respectively, from baseline.^40^, ^41^, ^42^, ^43^ Neutrophil count, C‐reactive protein (CRP) levels, and serum albumin levels were assessed. The upper limit of normal (ULN) for neutrophils was 7.23 × 10^9^/L and for CRP it was 10 mg/L; the lower limit of normal (LLN) for serum albumin was 38 g/L. Safety was assessed based on incidence and severity of adverse events (AEs).

### 2.3 Statistical analysis

All endpoints were assessed in patients who self‐reported as being Asian. The primary and key secondary endpoints were assessed for the randomized intention‐to‐treat (ITT) population with an exact Suissa–Shuster Z‐pooled test as previously described.^39^ The primary estimand used nonresponder imputation, with any values post death or any other medication for GPP representing nonresponse. Data presented for other endpoints are for the spesolimab group only (first dose plus optional second dose after Week 1 if flare symptoms persisted) because, consistent with the study design, most patients in the placebo group crossed over to the spesolimab group at Day 8, making comparisons versus placebo less useful over the 12‐week study. ITT observed analyses were carried out (including all values regardless of use of spesolimab or use of any other medication for GPP). The analysis for other endpoints includes observed values post open‐label spesolimab at Day 8; for markers of systemic inflammation, any values post use of open‐label spesolimab or any other medication for GPP were censored whereas for other endpoints they were imputed as the worst outcome in the calculation of median and quartiles. All data are summarized descriptively.

### 2.4 Ethics

The study was conducted in accordance with the study protocol, the International Council for Harmonization Good Clinical Practice guidelines, Regulation (EU) No. 536/2014, the Japanese Good Clinical Practice regulations, and applicable local regulations, and was approved by ethics committees of the participating institutions and countries. All the participating patients provided written informed consent.

## 3 RESULTS

### 3.1 Study participants

**FIGURE 1:**
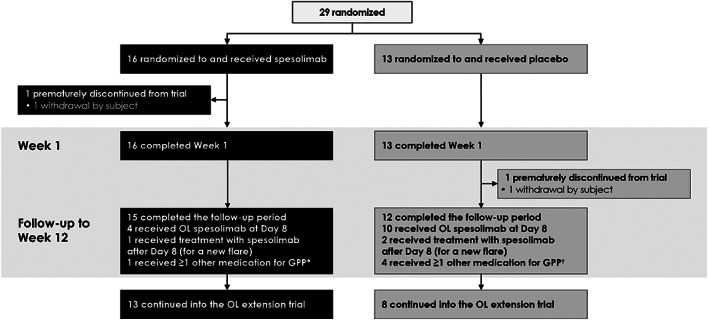
Patient disposition. *Cyclosporine, betamethasone butyrate propionate (*n* = 1); ^†^Ixekizumab (*n* = 2), adalimumab (*n* = 1), difluprednate (*n* = 1), and the following all taken by one patient prior to Week 1: cyclosporine, betamethasone dipropionate, betamethasone valerate, methotrexate, and prednisolone. GPP, generalized pustular psoriasis; OL, open‐label.

In total, 29 Asian patients were enrolled and randomized in the Effisayil™ 1 study, of whom 16 were randomized to spesolimab and 13 to placebo (Table 1 and Figure 1). The 12‐week follow‐up period was completed by 15 patients in the spesolimab group and 12 patients in the placebo group. All three patients in the spesolimab group with *IL36RN* mutations originated from Taiwan, while those in the placebo group originated from China (*n* = 2), Taiwan (*n* = 1), and Thailand (*n* = 1). Baseline demographics and clinical characteristics were generally comparable between the study groups (Table 1), with the exception of a greater proportion of women in the placebo group, a difference in distribution of GPPGA total and pustulation scores, and worse pain VAS and FACIT‐Fatigue scores in the spesolimab group. Baseline characteristics were also generally similar to those of the overall study population, except that, on average, Asian patients had lower body weight and BMI, lower Generalized Pustular Psoriasis Area and Severity Index total scores, and lower Japanese Dermatological Association Generalized Pustular Psoriasis Severity Index scores (Supporting Information Table S1).

**TABLE 1:** Baseline demographic and clinical characteristics of Asian patients in the Effisayil™ 1 study

Characteristic	Spesolimab (*n* = 16)	Placebo (*n* = 13)
Mean age (SD), years	42.2 (11.6)	43.2 (9.2)
Mean body weight (SD), kg	68.1 (18.1)	64.0 (19.0)
Mean BMI (SD), kg/m^2^	26.5 (6.2)	25.3 (7.6)
Female, *n* (%)	10 (62.5)	12 (92.3)
Country of enrollment, *n* (%)		
China	2 (12.5)	4 (30.8)
Japan	1 (6.3)	1 (7.7)
Malaysia	8 (50.0)	4 (30.8)
Singapore	0 (0.0)	1 (7.7)
Thailand	0 (0.0)	1 (7.7)
Taiwan	3 (18.8)	2 (15.4)
France	2 (12.5)	0 (0.0)
Present or past psoriasis, *n* (%)^a^ ^,^ ^b^	12 (75.0)	10 (76.9)
Ongoing plaque psoriasis, *n* (%)^2^	4 (25.0)	2 (15.4)
GPPGA total score, *n* (%)		
3	11 (68.8)	13 (100.0)
4	5 (31.3)	0 (0.0)
GPPGA pustulation subscore, *n* (%)		
2	3 (18.8)	4 (30.8)
3	7 (43.8)	6 (46.2)
4	6 (37.5)	3 (23.1)
Median GPPASI total score (Q1, Q3)	19.2 (14.8, 42.8)	19.4 (12.0, 28.7)
Median JDA‐GPPSI severity score (Q1, Q3)^c^	6.5 (4.0, 9.0)	7.5 (5.5, 9.5)
*IL36RN* mutation, *n* (%)^d^	3 (18.8)	4 (30.8)
*CARD14* mutation, *n* (%)^d^	0	0
*AP1S3* mutation, *n* (%)^d^	0	0
PRO scale, median (Q1, Q3)		
Pain VAS	77.1 (64.7, 84.2)	55.9 (44.9, 74.0)
FACIT‐Fatigue	15.5 (12.0, 27.5)	23.0 (17.0, 33.0)
DLQI	19.5 (15.5, 25.5)	16.0 (13.0, 22.0)
PSS	11.0 (10.0, 12.5)	10.0 (9.0, 11.0)
Body temperature, *n* (%)		
≤38.5°C	15 (93.8)	13 (100.0)
>38.5°C	1 (6.3)	0
CRP level, *n* (%)^c^		
<3 mg/L	1 (6.3)	1 (7.7)
≥3 to <70 mg/L	9 (56.3)	9 (69.2)
≥70 mg/L	5 (31.3)	2 (15.4)
WBC count, *n* (%)		
<10 × 10^9^/L	9 (56.3)	5 (38.5)
≥10 to <15 × 10^9^/L	5 (31.3)	7 (53.8)
≥15 × 10^9^/L	2 (12.5)	1 (7.7)
Neutrophil count, *n* (%)		
≤ULN (7.23 × 10^9^/L)	9 (56.3)	5 (38.5)
>ULN (7.23 × 10^9^/L)	7 (43.8)	8 (61.5)
Albumin level, *n* (%)^e^		
≥38 g/L	11 (68.8)	11 (84.6)
30 to <38 g/L	3 (18.8)	1 (7.7)
<30 g/L	1 (6.3)	0
Hospitalized for current GPP flare, *n* (%)	5 (31.3)	6 (46.2)
Number of days in hospital for current GPP flare, median (Q1, Q3)	8.0 (7.0, 8.0)^f^	6.5 (4.0, 11.0)^g^
Number of days in ICU for current GPP flare, median (Q1, Q3)	0 (0, 0)^f^	0 (0, 0)^g^

Abbreviations: BMI, body mass index; CRP, C‐reactive protein; DLQI, dermatology quality of life index; FACIT‐Fatigue, functional assessment of chronic illness therapy–fatigue; GPP, generalized pustular psoriasis; GPPASI, Generalized Pustular Psoriasis Area and Severity Index; GPPGA, Generalized Pustular Psoriasis Physician Global Assessment; ICU, intensive care unit; JDA‐GPPSI, Japanese Dermatological Association Generalized Pustular Psoriasis Severity Index; PRO, patient‐reported outcome; PSS, psoriasis symptom scale; Q, quartile; SD, standard deviation; ULN, upper limit of normal; VAS, visual analog scale; WBC, white blood cell.

^a^ Data are missing for one participant in the spesolimab group.

^b^ ‘Present or past psoriasis’ could encompass any type of psoriasis including plaque psoriasis, while ‘ongoing plaque psoriasis’ refers exclusively to plaque psoriasis.

^c^ Data are missing for one participant in the placebo group.

^d^ DNA sequencing was not performed in three participants in the spesolimab group and one in the placebo group.

^e^ Data are missing for one participant in the spesolimab group and one in the placebo group.

^f^ *n* = 5.

^g^ *n* = 6.

In the spesolimab group, four patients received an open‐label dose of spesolimab at Day 8 for persistent flare symptoms; of these, one patient received additional spesolimab after Day 8 (at Day 58) for a new flare. In the placebo group, 10 patients received an open‐label dose of spesolimab at Day 8 for persistent flare symptoms; two of these patients received additional spesolimab after Day 8 (at Days 44 and 68, respectively) for a new flare.

### 3.2 Efficacy

**FIGURE 2:**
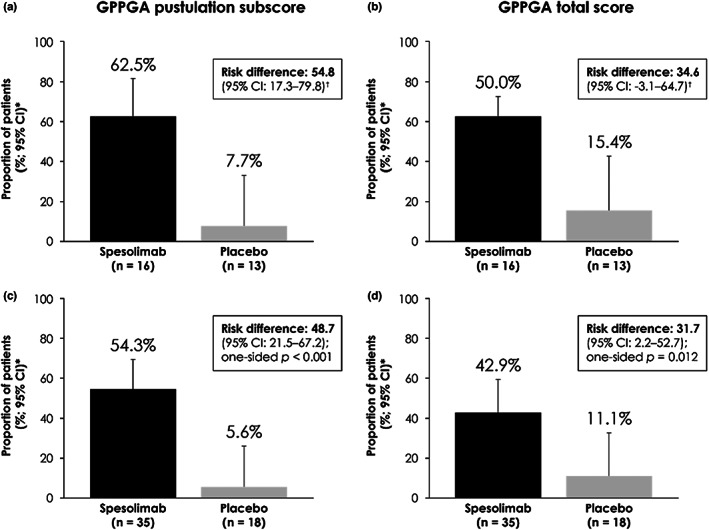
Proportion of Asian patients (a and b) and patients in the overall population of the Effisayil™ 1 study (c and d) with a GPPGA pustulation subscore of 0 (no visible pustules) and a GPPGA total score of 0 or 1 (clear or almost clear skin) at Week 1. Primary analysis: any values post death or any other medication for GPP represent nonresponse. *95% CIs calculated using the method of Wilson; ^†^95% CIs calculated using the method of Chan and Zhang. CI, confidence interval; GPP, generalized pustular psoriasis; GPPGA, Generalized Pustular Psoriasis Physician Global Assessment.

**FIGURE 3:**
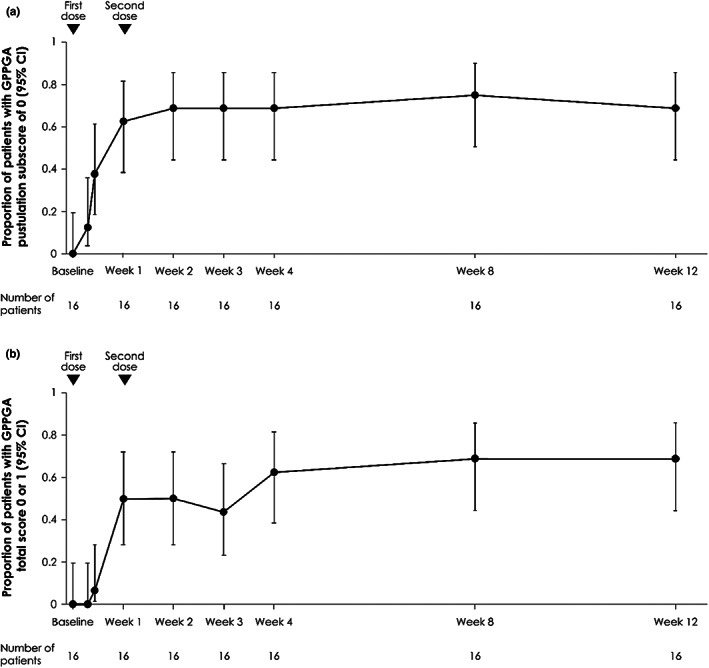
Proportion of patients in the spesolimab group with a GPPGA pustulation subscore of 0 (no visible pustules) (a) and a GPPGA total score of 0 or 1 (clear or almost clear skin) (b) up to week 12. Data are shown for patients initially randomized to receive spesolimab who received up to two doses of spesolimab: Day 1 (*n* = 16) and an optional OL dose at Day 8 (*n* = 4). Missing values, any use of other medication for GPP, or use of spesolimab for the treatment of a new GPP flare were regarded as nonresponse for this analysis. CI, confidence interval; GPP, generalized pustular psoriasis; GPPGA, Generalized Pustular Psoriasis Physician Global Assessment; OL, open‐label.

The primary endpoint, a GPPGA pustulation subscore of 0 (no visible pustules) at Week 1, was achieved by 10 patients (62.5%) in the spesolimab group and one patient (7.7%) in the placebo group (risk difference 54.8, 95% confidence interval [CI] 17.3–79.8; Figure 2a). The key secondary endpoint, a GPPGA total score of 0 or 1 (clear or almost clear skin) at Week 1, was achieved by eight patients (50.0%) in the spesolimab group and two patients (15.4%) in the placebo group (risk difference 34.6, 95% CI −3.1–64.7; Figure 2b). Corresponding data for the Effisayil™ 1 overall population are also shown (Figure 2c,d). Achievement of a GPPGA pustulation subscore of 0 was sustained in 68.8% of Asian patients over 12 weeks. Similarly, achievement of a GPPGA total score of 0 or 1 was also sustained in at least 68.8% of Asian patients over 12 weeks (Figure 3). Results were consistent when assessed according to the ITT principle; when patients randomized to placebo received open‐label spesolimab at Day 8, the proportion of patients achieving these scores rapidly increased (Supporting Information Figure S1).

Of the three patients with *IL36RN* mutations in the spesolimab group, two achieved the primary and key secondary endpoints. Of the four patients with *IL36RN* mutations in the placebo group, one achieved these endpoints. Of the 10 patients in the spesolimab group without such mutations, the primary and key secondary endpoints were achieved by six and four patients, respectively. In the placebo group, no patient without an *IL36RN* mutation achieved the primary endpoint and one patient achieved the key secondary endpoint.

In patients randomized to spesolimab, the Japanese Dermatological Association Generalized Pustular Psoriasis Severity Index improved over time, with a median (interquartile range [IQR] Q1, Q3) absolute change from baseline of −1.50 (−3.0, 0.0) at Week 1, which further reduced up to Week 4 and was sustained up to Week 12 (Figure S2).

### 3.3 Patient‐reported outcomes

**FIGURE 4:**
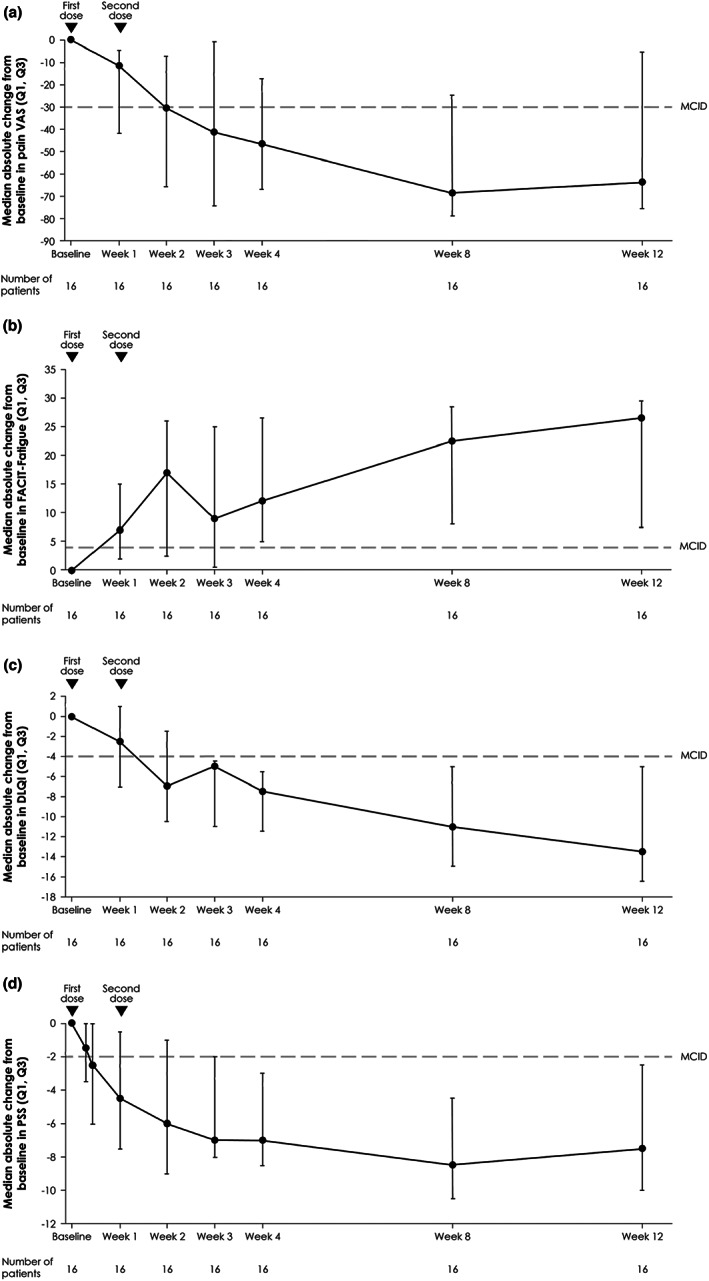
Median absolute changes from baseline over time in pain VAS (a), FACIT‐fatigue (b), DLQI (c), and PSS (d). Data are shown for patients initially randomized to receive spesolimab who received up to two doses of spesolimab: Day 1 (*n* = 16) and an optional OL dose at Day 8 (*n* = 4). Use of other medication for GPP or use of spesolimab for the treatment of a new GPP flare were regarded as worst outcome for this analysis. Missing data were imputed using the last‐observation‐carried‐forward method. The dashed line indicates the MCID thresholds: −30 points for pain VAS,^42^ +4 points for FACIT‐Fatigue,^41^ –4 points for DLQI,^40^ and −2 points for PSS.^43^ DLQI, Dermatology Life Quality Index; FACIT‐Fatigue, Functional Assessment of Chronic Illness Therapy–Fatigue; GPP, generalized pustular psoriasis; MCID, Minimal Clinically Important Difference; OL, open‐label; pain VAS, pain Visual Analog Scale; PSS, Psoriasis Symptom Scale; Q, quartile.

In patients randomized to spesolimab, continuous improvement was seen in pain VAS, FACIT‐Fatigue, DLQI, and PSS over 8 weeks, with some plateauing of the effect between Weeks 8 and 12 (Figure 4). Spesolimab treatment led to the achievement of MCIDs in all four PRO scales: FACIT‐Fatigue and PSS by Week 1 and pain VAS and DLQI by Week 2. This achievement of MCIDs was sustained up to Week 12, with median (IQR Q1, Q3) absolute changes from baseline of −63.5 (−75.45, −5.24), 26.5 (7.5, 9.5), −13.5 (−16.5, −5.0), and −7.5 (−10.0, −2.5) for pain VAS, FACIT‐Fatigue, DLQI, and PSS, respectively. All PRO scores surpassed the threshold for MCIDs at Week 12. When assessed according to the ITT principle, results were consistent. In a similar manner to that observed for GPPGA scores, PRO scores rapidly improved for patients randomized to placebo who subsequently received open‐label spesolimab at Day 8 (Figure S3).

### 3.4 Markers of systemic inflammation

**FIGURE 5:**
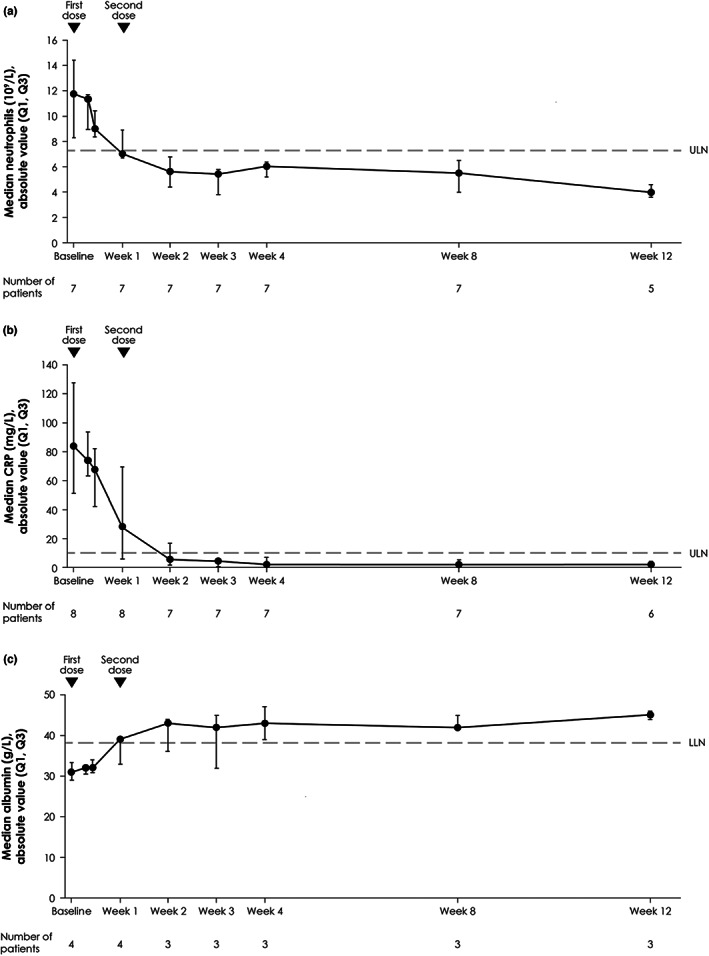
Median absolute values from baseline over time in neutrophil count (a), CRP levels (b), and serum albumin levels (c) for patients in the spesolimab group who had abnormal count/levels at baseline. Data are shown for patients initially randomized to receive spesolimab who received up to two doses of spesolimab. (a) Data for seven patients who had baseline neutrophil counts above the ULN of 7.23 × 10^9^/L. (b) Data for eight patients who had baseline CRP levels above the ULN of 10 mg/L. (c) Data for four patients with baseline serum albumin levels below the LLN of 38 g/L. All values after any use of other medication for GPP or use of spesolimab for the treatment of a new GPP flare are excluded. The dashed lines indicate the ULN or LLN. CRP, C‐reactive protein; GPP, generalized pustular psoriasis; LLN, lower limit of normal; Q, quartile; ULN, upper limit of normal.

In patients randomized to spesolimab and who had an elevated neutrophil count that was above ULN at baseline (7.23 × 10^9^/L, *n* = 7), median absolute values returned to below the ULN within 1 week. For patients with elevated CRP levels above the ULN at baseline (10 mg/L, *n* = 8), values returned to below the ULN within 2 weeks, and there was a consistent decrease in levels over the 12 weeks following treatment with spesolimab (Figure 5). For patients with low serum albumin levels below the LLN (38 g/L, *n* = 4), median absolute values returned to above the LLN within 1 week; this was sustained throughout the study (Figure 5). For patients treated with spesolimab, elevated baseline neutrophil counts, elevated CRP levels, and low serum albumin levels had normalized at Week 2 in 6/7, 5/7, and 2/3 patients, respectively.

### 3.5 Safety

By Week 1, 11 patients (68.8%) in the spesolimab group and eight patients (61.5%) in the placebo group had reported at least one AE (Table 2). Pyrexia and dizziness were more frequently reported in the placebo group than in the spesolimab group. There were two cases of urinary tract infection in the spesolimab group compared with none in the placebo group. One patient experienced three serious AEs by Week 1 (urinary tract infection and symptoms that were reported as drug‐induced liver injury and drug reaction with eosinophilia and systemic symptoms [DRESS]). In this patient, the onset of symptoms occurred 2 days after spesolimab treatment initiation, and resolution occurred within 10 days. Because the accepted features of DRESS include onset and resolution occurring ≥14 days after treatment administration,^44^ a causal relationship between spesolimab and the AE was considered implausible. According to the European Registry of Severe Cutaneous Adverse Reactions (RegiSCAR) scoring system, a score of 1 (“no DRESS”) was assigned to this patient. Through Week 12, 20 patients (74.1%) who received at least one dose of spesolimab reported at least one AE. No deaths were reported during the study.

**TABLE 2:** Adverse events occurring in Asian patients in the Effisayil™ 1 study

*n* (%) [rate/100 patient‐years]	Week 1		Week 12^a^
	Spesolimab (*n* = 16)	Placebo (*n* = 13)	Spesolimab (*n* = 27)
Any AE	11 (68.8) [6809.7]	8 (61.5) [5513.2]	20 (74.1) [812.6]
Common AE^b^			
Urinary tract infection	2 (12.5) [716.2]	0 (0.0) [0.0]	2 (7.4) [32.5]
Headache	1 (6.3) [344.6]	1 (7.7) [419.8]	3 (11.1) [48.8]
Pain in extremity	1 (6.3) [347.9]	1 (7.7) [419.8]	3 (11.1) [47.5]
Diarrhea	1 (6.3) [338.2]	0 (0.0) [0.0]	3 (11.1) [47.8]
Pyrexia	1 (6.3) [344.6]	3 (23.1) [1461.0]	3 (11.1) [47.9]
Peripheral edema	2 (12.5) [695.7]	1 (7.7) [419.8]	2 (7.4) [32.1]
Dizziness	0 (0.0) [0.0]	2 (15.4) [880.1]	3 (11.1) [48.8]
Nausea	1 (6.3) [341.4]	0 (0.0) [0.0]	2 (7.4) [30.7]
Severe AE (RCTC grade 3 or 4)	0 (0.0) [0.0]	1 (7.7) [429.7]	1 (3.7) [14.9]
Investigator‐defined drug‐related AE	5 (31.3) [1963.7]	5 (38.5) [2685.7]	12 (44.4) [258.4]
Serious AE	1 (6.3) [351.2]	0 (0.0) [0.0]	2 (7.4) [31.2]
Urinary tract infection	1 (6.3) [351.2]	0 (0.0) [0.0]	1 (3.7) [15.4]
DILI	1 (6.3) [351.2]	0 (0.0) [0.0]	1 (3.7) [15.4]
DRESS	1 (6.3) [351.2]	0 (0.0) [0.0]	1 (3.7) [15.4]
Squamous cell carcinoma of the skin	0 (0.0) [0.0]	0 (0.0) [0.0]	1 (3.7) [14.9]
Serious AE resulting in death	0 (0.0) [0.0]	0 (0.0) [0.0]	0 (0.0) [0.0]
Serious AE requiring or prolonging hospitalization	1 (6.3) [351.2]	0 (0.0) [0.0]	1 (3.7) [15.4]

Abbreviations: AE, adverse event; DILI, drug‐induced liver injury; DRESS, drug reaction with eosinophilia and systemic symptoms; RCTC, Rheumatology Common Toxicity Criteria.

^a^ Shown are the number of AEs occurring between the start of treatment and the end of the residual effect period. For patients who received optional spesolimab on or after Day 8, the residual effect period was 16 weeks after the last dose of study medication. AEs occurring during the residual effect period were considered “treatment‐emergent” and coded using the Medical Dictionary for Drug Regulatory Activities version 23.1.

^b^ AEs reported in ≥10% of patients in any group. AE severity was graded according to the RCTC version 2.0 safety analysis set. Pustular psoriasis was excluded as an AE from this safety analysis.

## 4 DISCUSSION

In the Effisayil™ 1 study of patients with a GPP flare, spesolimab treatment provided rapid and sustained improvements in GPPGA pustulation subscore and GPPGA total score compared with placebo, with an acceptable safety profile.^39^ Among the Asian participants, baseline demographics and clinical characteristics were comparable between study groups; they were also broadly similar to the overall population but with some differences, such as lower body weight and BMI in Asian patients than the overall population. This finding is consistent with another clinical trial of Asian patients.^45^ Additionally, in this study, rates of present/past and ongoing plaque psoriasis were similar between Asian patients, non‐Asian patients, and the overall population (Supporting Information Table S1); this is in contrast with previous literature, which suggests a lower rate in Asia than Europe.^46^ Rates of *IL36RN* mutations were comparable between Asian patients and the overall population, and there was no clear evidence that these mutations affected the efficacy of spesolimab; however, genotyping data were not available for every patient. The efficacy of spesolimab in the overall population has previously been shown to remain unaffected by *IL36RN* mutations.^47^

The efficacy of spesolimab in Asian patients was also comparable to that in the overall population, with spesolimab providing a treatment benefit compared with placebo at Week 1 in terms of achievement of the primary and key secondary endpoints, and through sustained reductions in GPPGA scores up to Week 12. The percentages of Asian patients achieving a GPPGA pustulation subscore of 0 and a GPPGA total score of 0 or 1 at Week 1 (63% and 50%, respectively) were slightly higher than in the overall population (54% and 43%). However, caution must be taken when interpreting these results because no formal statistical comparisons were made; any numerical differences could be chance findings. Spesolimab also improved pain, fatigue, overall quality of life, and cutaneous symptoms in Asian patients, as assessed by PRO scales, and normalized markers of systemic inflammation. Such findings are in agreement with the improvements seen in the clinician‐reported GPPGA; a reduction in disease severity would be expected to lead to improvements in patient experience of GPP and a reduction in overall inflammation. When the GPPGA scores and PRO scales were analyzed according to the ITT principle, in which any use of open‐label spesolimab or other medication was included, improvements were seen over time, consistent with the primary results.^39^ Additionally, once patients randomized to receive placebo received optional open‐label spesolimab at Day 8, rapid improvements in outcomes were observed. Safety was comparable to the overall population, with no increased risk of serious infections. One patient had a serious AE reported as DRESS; however, as previously noted, this was not considered related to spesolimab.

These results compare favorably to those of other biologics studied for the treatment of GPP in Asian patients. Currently available treatments for GPP in Asia have displayed efficacy in GPP; however, as noted, the studies reporting these treatments are often open label and single arm or have enrolled fewer than 12 patients.^2^, ^25^, ^26^, ^29^, ^48^, ^49^ In contrast, Effisayil™ 1 was an adequately powered, randomized, placebo‐controlled trial with sufficient patient numbers to assess efficacy and safety in population subgroups. Moreover, the results presented here are the first data for a GPP treatment administered to Asian patients selected according to the occurrence of a flare.

The limitations of this study include the small number of patients enrolled and the even fewer patients in the subgroup analysis. No formal statistical comparisons could be made, meaning that caution is required when interpreting the findings. This is particularly the case for analyses involving fewer patients than the whole Asian population in the study; for example, although 67% and 60% of patients with and without *IL36RN* mutations achieved the primary endpoint, respectively, the denominators in these groups are small and therefore limit interpretation. However, as previously noted, GPP is a rare disease and therefore performing trials with a large number of participants is impractical. Effisayil™ 1 studied a larger Asian population than any other GPP study yet reported, and sufficient patients were recruited to measure outcomes. Another limitation of the study was that most patients in the placebo group crossed over to the spesolimab group at Day 8, although analyses of efficacy that excluded receipt of spesolimab after Day 8 showed that spesolimab improved outcomes over time.

In conclusion, spesolimab treatment improved outcomes in Asian patients compared with placebo, supporting its use in the treatment of GPP flares.

## FUNDING INFORMATION

The study was supported and funded by Boehringer Ingelheim.

## DISCLOSURES

Akimichi Morita declares receiving research grants, consulting fees, and/or speaker's fees from AbbVie, Boehringer Ingelheim, Eisai, Eli Lilly, Janssen, Kyowa Kirin, LEO Pharma, Maruho, Mitsubishi Tanabe, Nichi‐Iko, Nippon Kayaku, Novartis, Sun Pharmaceutical Industries, Taiho Pharmaceutical, Torii Pharmaceutical, and Ushio. Tsen‐Fang Tsai declares conducting clinical trials or paid consulting activities for AbbVie, Boehringer Ingelheim, Celgene, Eli Lilly, GSK, Janssen, MSD, Novartis, Pfizer, and UCB. Evelyn Yap Wen Yee declares being an investigator for AbbVie, AnaptysBio, Boehringer Ingelheim, La Roche‐Posay, and Novartis, and a consultant for AnaptysBio. Yukari Okubo declares receiving grants or contracts from Eisai, Maruho, and Torii Pharmaceutical, and consulting fees from AbbVie, Amgen, Boehringer Ingelheim, Bristol Myers Squibb, Celgene, Eisai, Eli Lilly, Janssen, JIMRO, Kyowa Kirin, LEO Pharma, Maruho, Novartis, Pfizer, Sanofi, Sun Pharmaceutical Industries, Taiho Pharmaceutical, Mitsubishi Tanabe, Torii Pharmaceutical, and UCB. Shinichi Imafuku has served as a consultant and/or paid speaker for and/or accepted a research grant from and/or participated in clinical trials sponsored by companies including AbbVie, Amgen, Eisai, Eli Lilly, Janssen, Kyowa Kirin, LEO Pharma, Maruho, Mitsubishi Tanabe, Novartis, Sun Pharmaceutical Industries, Taiho Pharmaceutical, Torii Pharmaceutical, and UCB. Min Zheng declares receiving grants, consulting fees, and/or speaker's fees from AbbVie, Boehringer Ingelheim, Janssen, LEO Pharma China, Novartis, Pfizer, and Xian‐Janssen. Ling Li, Manuel Quaresma, and Christian Thoma are employees of Boehringer Ingelheim. Siew Eng Choon declares paid activities as an advisor, speaker, or consultant for AbbVie, Boehringer Ingelheim, Eli Lilly, Janssen, LEO Pharma, MSD, Novartis, Pfizer, Sanofi, and UCB. The authors met criteria for authorship as recommended by the International Committee of Medical Journal Editors (ICMJE). The authors did not receive payment related to the development of this manuscript. Boehringer Ingelheim was given the opportunity to review the manuscript for medical and scientific accuracy as well as intellectual property considerations.

## Supporting information


Data S1

## Data Availability

To ensure independent interpretation of clinical study results and enable authors to fulfill their role and obligations under the ICMJE criteria, Boehringer Ingelheim grants all external authors access to clinical study data pertinent to the development of the publication. In adherence with the Boehringer Ingelheim Policy on Transparency and Publication of Clinical Study Data, scientific and medical researchers can request access to clinical study data when it becomes available on Vivli ‐ Center for Global Clinical Research Data (https://eur03.safelinks.protection.outlook.com/?url=https%3A%2F%2Fvivli.org%2F&data=05%7C01%7Cswati.krishnan%40boehringer‐ingelheim.com%7C1f1372e19ab449feaf5508da716f0191%7Ce1f8af86ee954718bd0d375b37366c83%7C0%7C0%7C637947018824415984%7CUnknown%7CTWFpbGZsb3d8eyJWIjoiMC4wLjAwMDAiLCJQIjoiV2luMzIiLCJBTiI6Ik1haWwiLCJXVCI6Mn0%3D%7C3000%7C%7C%7C&sdata=%2FlCnIi95EokaW4hoIC0eR5w57gU1bgC7cm%2BVvWlJr3A%3D&reserved=0), and earliest after publication of the primary manuscript in a peer‐reviewed journal, regulatory activities are complete, and other criteria are met. Please visit Medical & Clinical Trials | Clinical Research | MyStudyWindow (https://eur03.safelinks.protection.outlook.com/?url=https%3A%2F%2Fwww.mystudywindow.com%2Fmsw%2Fdatasharing&data=05%7C01%7Cswati.krishnan%40boehringer‐ingelheim.com%7C1f1372e19ab449feaf5508da716f0191%7Ce1f8af86ee954718bd0d375b37366c83%7C0%7C0%7C637947018824415984%7CUnknown%7CTWFpbGZsb3d8eyJWIjoiMC4wLjAwMDAiLCJQIjoiV2luMzIiLCJBTiI6Ik1haWwiLCJXVCI6Mn0%3D%7C3000%7C%7C%7C&sdata=%2FK8Fl%2Be3G4xnN5jh38nwo66DHWfsxxjdTdPllp3AhJw%3D&reserved=0) for further information.
