# Supplementary material for: Efficacy and safety of spesolimab in Asian patients with a generalized pustular psoriasis flare: Results from the randomized, double‐blind, placebo‐controlled Effisayil™ 1 study
Source: J Dermatol. 2022 Oct 25;50(2):183–94. doi: 10.1111/1346-8138.16609 (PMC10092680; doi:10.1111/1346-8138.16609)
Supplement: Supplementary file 1 — Data S1 [file JDE-50-183-s001.docx]

**Efficacy and safety of spesolimab in Asian patients with a generalized pustular psoriasis flare: Results from the randomized, double-blind, placebo-controlled Effisayil™ 1 study**

Akimichi Morita, Tsen-Fang Tsai, Evelyn Yap Wen Yee, Yukari Okubo, Shinichi Imafuku, Min Zheng, Ling Li, Manuel Quaresma, Christian Thoma, Siew Eng Choon

**Supplementary figures and tables**

**Figure S1.** Proportion of patients with GPPGA pustulation subscore of 0 (no visible pustules) (A) and GPPGA total score of 0 or 1 (clear or almost clear skin) (B) over time (intention-to-treat analysis)


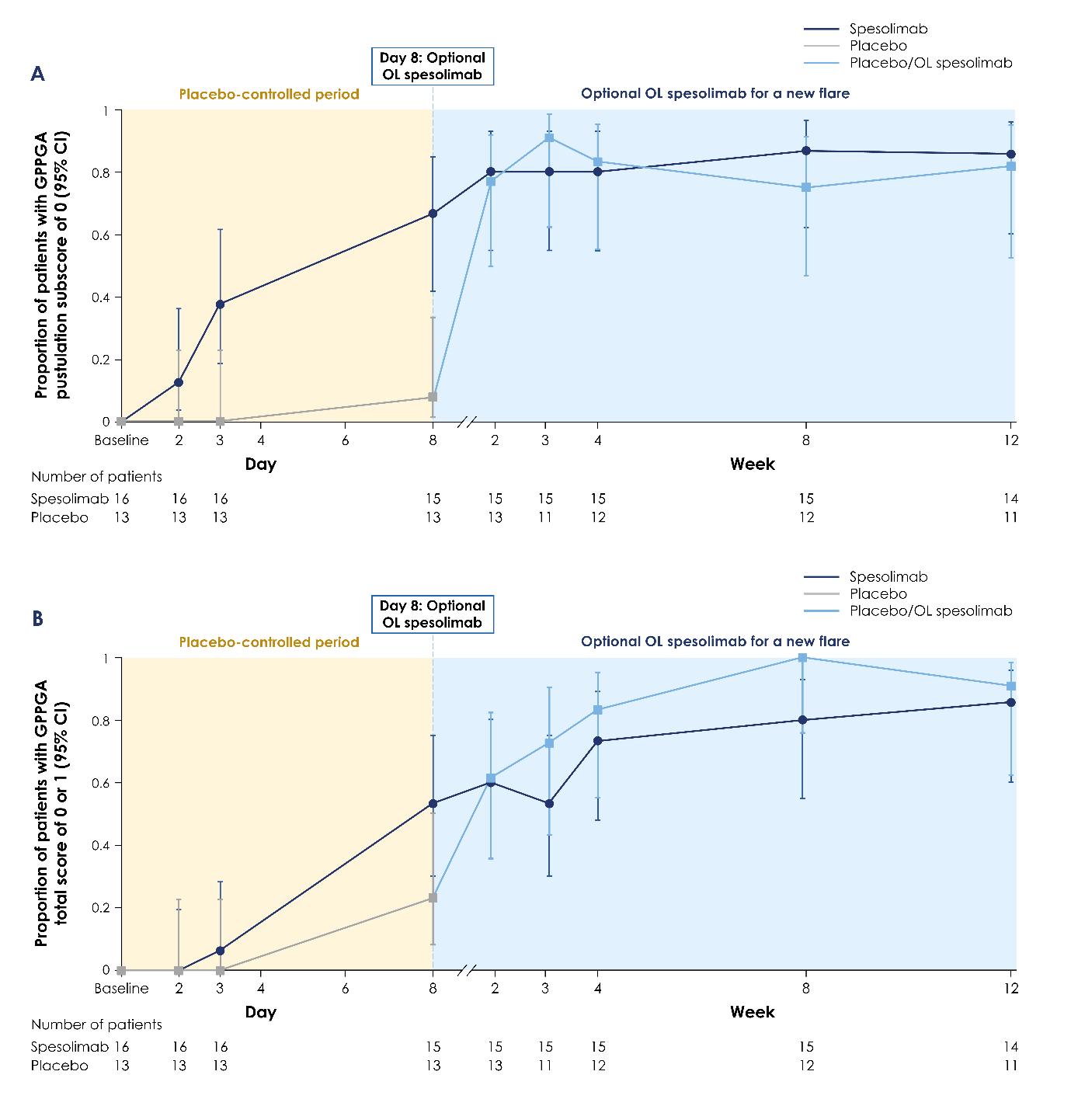


Data are all observed cases regardless of use of any other medication for GPP or any additional dose of spesolimab. At Day 8, four patients randomized to the spesolimab group and 10 patients randomized to the placebo group received OL spesolimab. After Day 8, one patient in the spesolimab group and two in the placebo group received spesolimab for a new flare.

CI, confidence interval; GPP, generalized pustular psoriasis; GPPGA, Generalized Pustular Psoriasis Physician Global Assessment; OL, open-label.

**Figure S2.** Absolute change from baseline in JDA-GPPSI up to Week 12


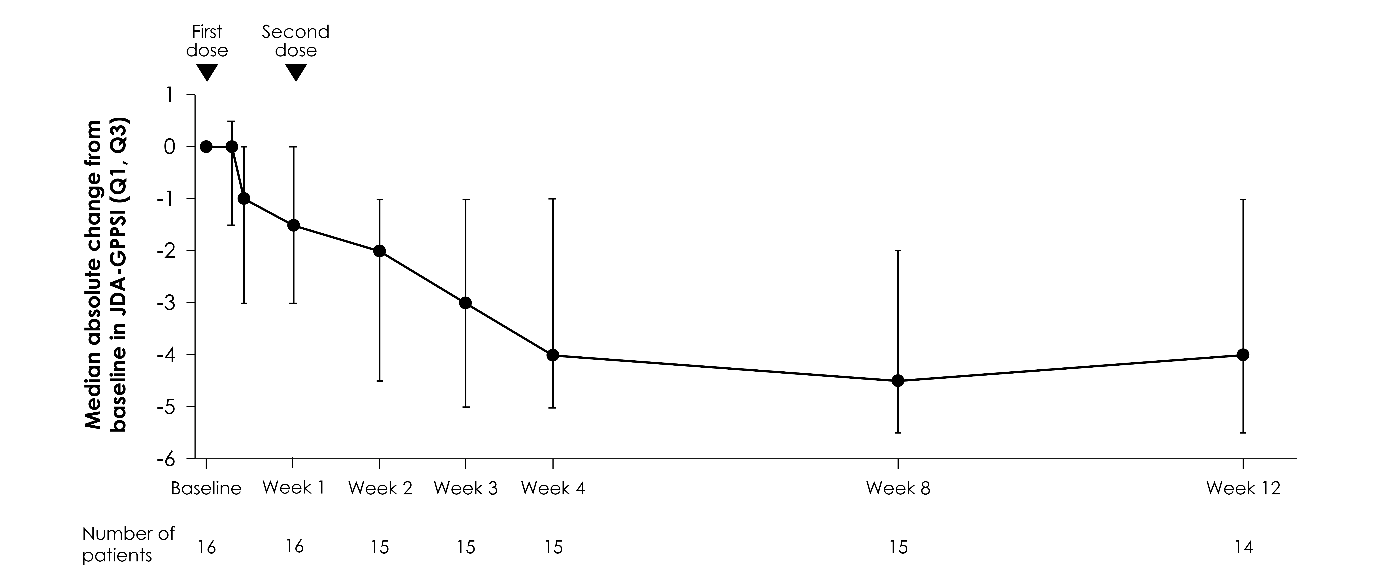


Treatment effect in patients initially randomized to receive spesolimab who received up to two doses of spesolimab: Day 1 (n = 16) and an optional OL dose at Day 8 (n = 4). Missing values, any use of other medication for GPP, or use of spesolimab for the treatment of a new GPP flare were regarded as worst outcome for this analysis.

GPP, generalized pustular psoriasis; JDA-GPPSI, Japanese Dermatological Association Generalized Pustular Psoriasis Severity Index; OL, open-label; Q, quartile.

**Figure S3.** Mean absolute changes from baseline over time in pain VAS (A), FACIT-Fatigue (B), DLQI (C), and PSS (D).


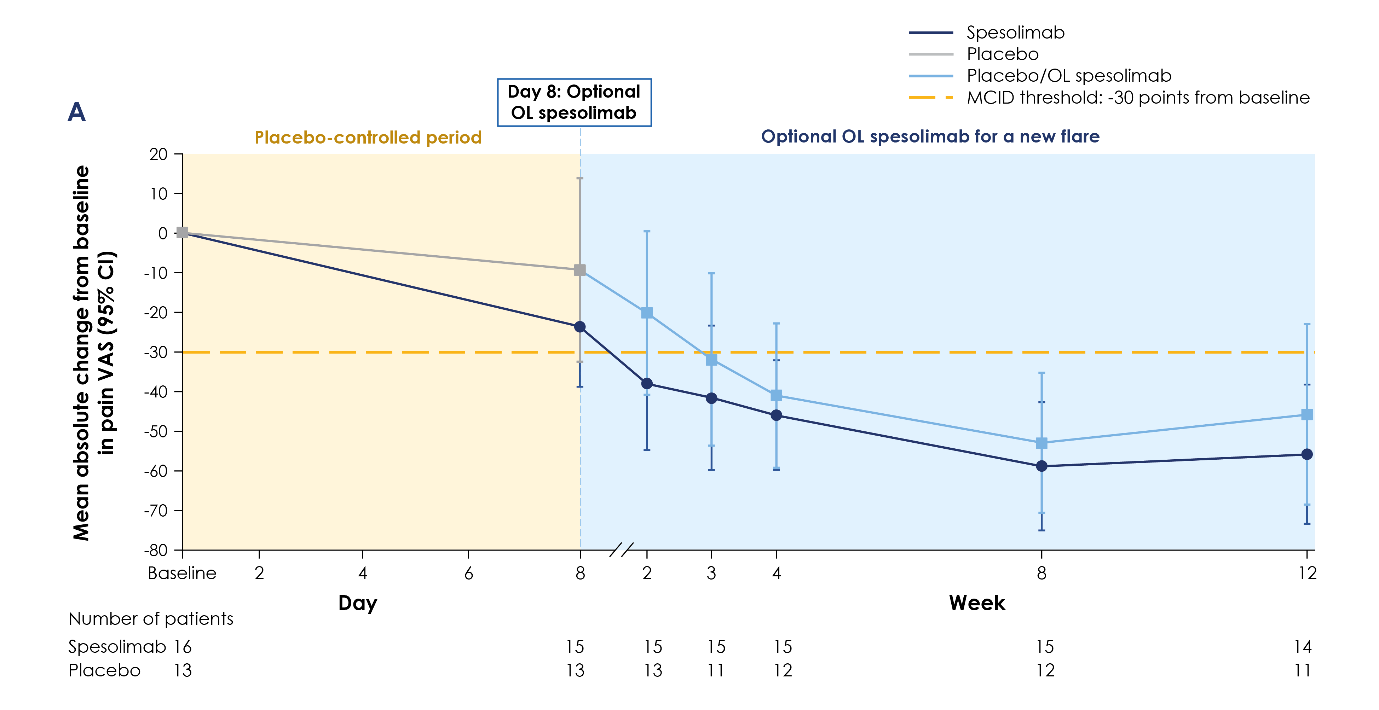


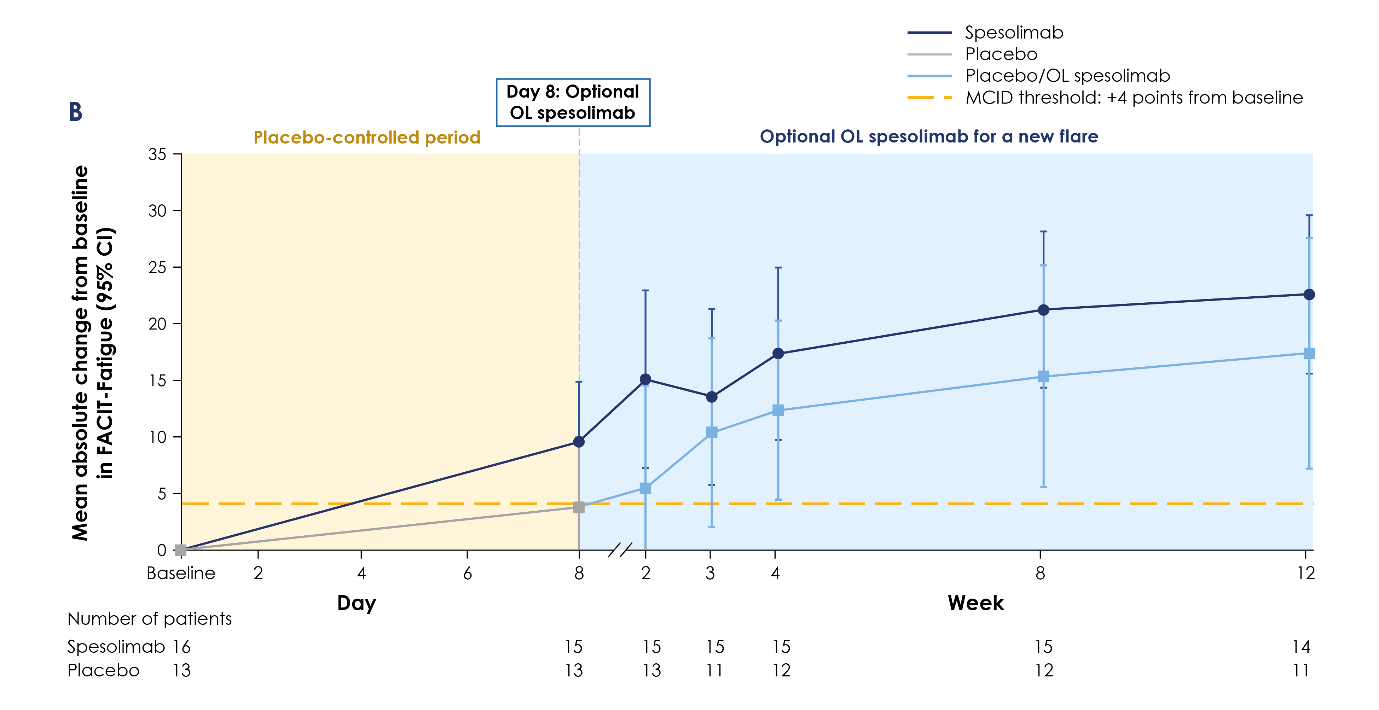


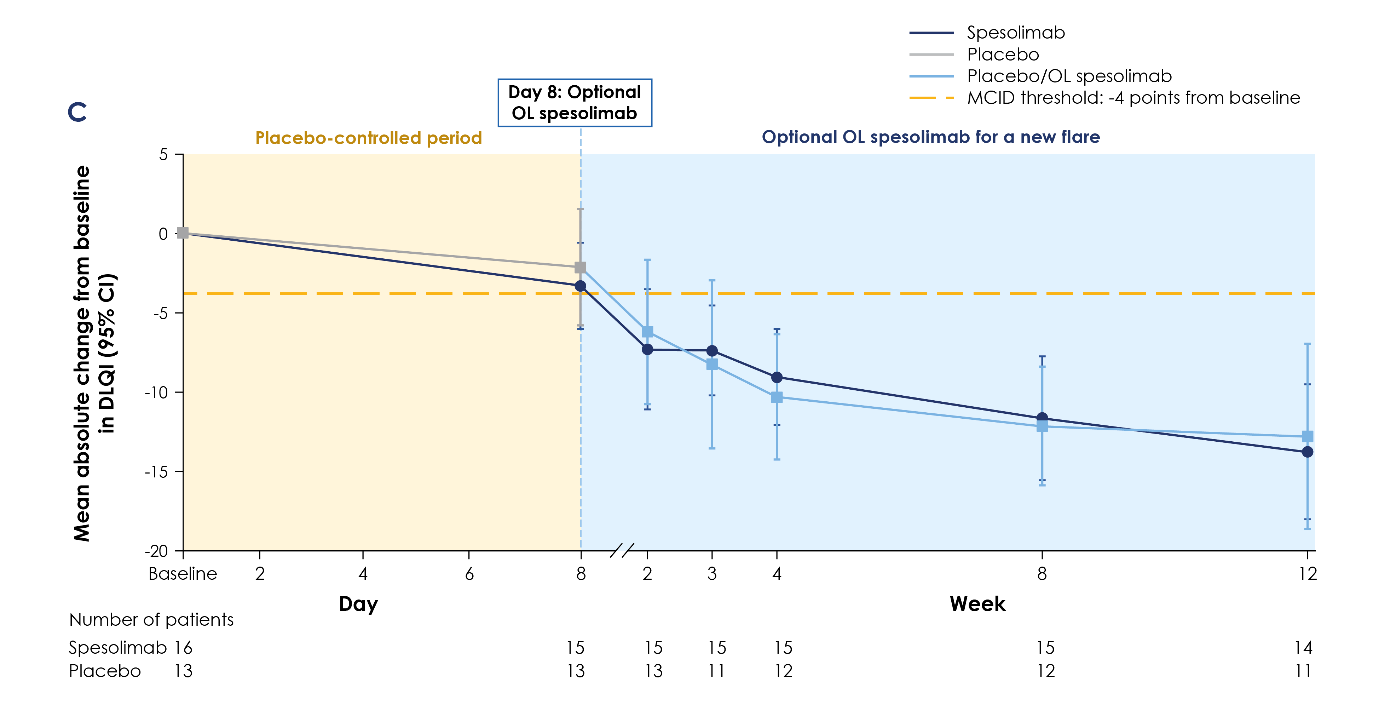


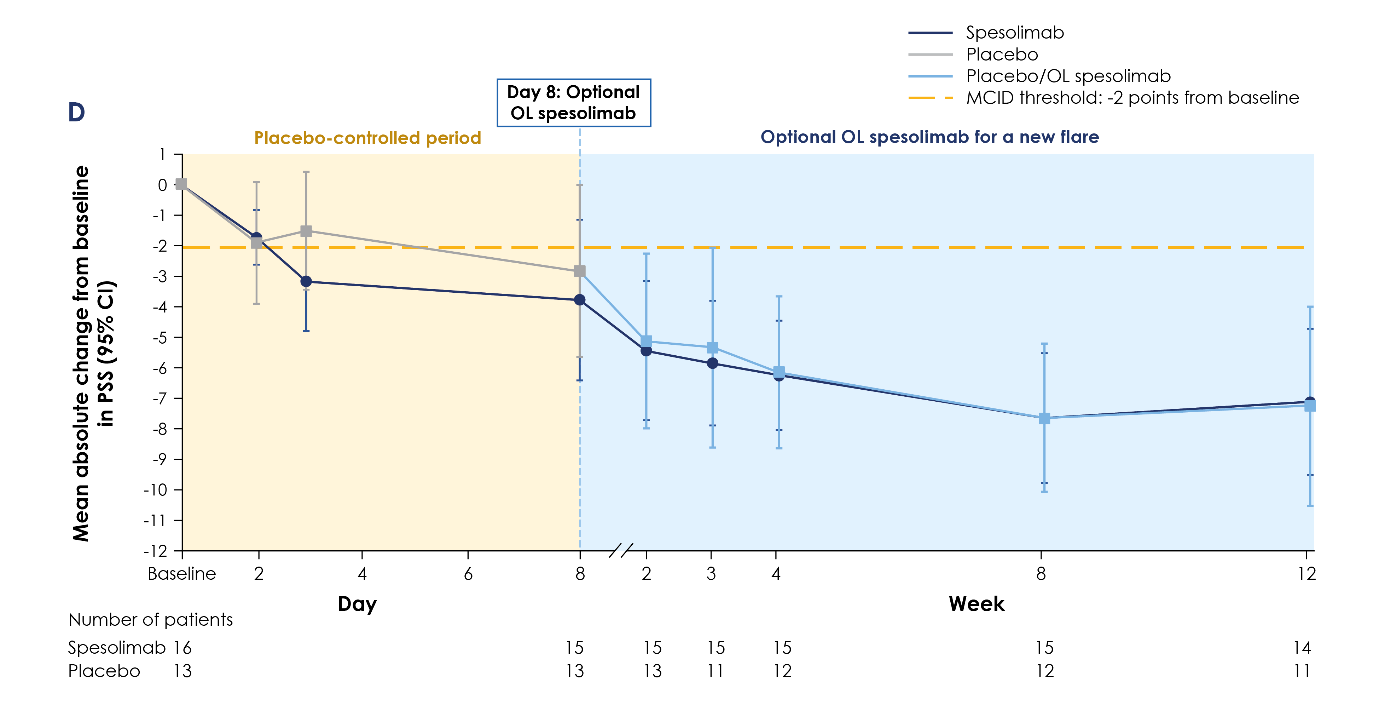


Data are all observed cases regardless of use of any other medication for GPP or any additional dose of spesolimab. At Day 8, four patients randomized to the spesolimab group and 10 patients randomized to the placebo group received OL spesolimab. After Day 8, one patient in the spesolimab group and two in the placebo group received spesolimab for a new flare. The dashed line indicates the MCID thresholds: −30 points for pain VAS,^41^ +4 points for FACIT-Fatigue,^40^ −4 points for DLQI,^39^ and −2 points for PSS.^42^

CI, confidence interval; DLQI, Dermatology Life Quality Index; FACIT-Fatigue, Functional Assessment of Chronic Illness Therapy–Fatigue; GPP, generalized pustular psoriasis; MCID, minimal clinically important difference; OL, open-label; pain VAS, pain visual analog scale; PSS, Psoriasis Symptom Scale.

**Table S1**. Baseline demographics and clinical characteristics of Asian patients, non-Asian patients, and the overall study population in the Effisayil^TM^ 1 study

| **Characteristic** | **Asian patients**  **(n = 29)** | **Non-Asian patients**  **(n = 24)** | **Overall study population (n = 53)** |
| --- | --- | --- | --- |
| Mean age (SD), years | 42.6 (10.4) | 43.5 (11.7) | 43.0 (10.9) |
| Mean body weight (SD), kg | 66.3 (18.3) | 78.99 (29.67) | 72.0 (24.7) |
| Mean BMI (SD), kg/m^2^ | 26.0 (6.8) | 28.21 (9.83) | 27.0 (8.3) |
| Female, n (%) | 22 (75.9) | 14 (58.3) | 36 (67.9) |
| Pooled study site, n (%) |  |  |  |
| USA | 0 | 3 (12.5) | 3 (5.7) |
| Japan | 2 (6.9) | 0 | 2 (3.8) |
| Asia (excluding Japan) | 25 (86.2) | 0 | 25 (47.2) |
| Europe | 2 (6.9) | 14 (58.3) | 17 (32.1) |
| Africa | 0 | 7 (29.2) | 7 (13.2) |
| Present or past psoriasis, n (%) | 22 (75.9)^†^ | 16 (66.7) | 38 (71.2) |
| Ongoing plaque psoriasis, n (%) | 6 (20.7) | 3 (12.5) | 9 (17.0) |
| GPPGA total score, n (%) |  |  |  |
| 3 | 24 (82.8) | 19 (79.2) | 43 (81.1) |
| 4 | 5 (17.2) | 5 (20.8) | 10 (18.9) |
| GPPGA pustulation subscore, n (%) |  |  |  |
| 2 | 7 (24.1) | 4 (16.7) | 11 (20.8) |
| 3 | 13 (44.8) | 10 (41.7) | 23 (43.4) |
| 4 | 9 (31.0) | 10 (41.7) | 19 (35.8) |
| Median GPPASI total score (Q1, Q3) | 19.4 (14.3, 32.1) | 30.6 (19.2, 36.2) | 27.2 (15.4, 36.1) |
| Median JDA-GPPSI severity score (Q1, Q3) | 7.0 (5.0, 9.5)^‡^ | 9.0 (8.0, 10.0)^§^ | 8.0 (6.0, 10.0) |
| *IL36RN* mutation, n (%) | 7 (24.1)^¶^ | 7 (29.2)^††^ | 14 (26.4) |
| *CARD14* mutation, n (%) | 0^¶^ | 5 (20.8)^††^ | 5 (9.4) |
| *AP1S3* mutation, n (%) | 0^¶^ | 1 (4.2)^††^ | 1 (1.9) |
| PRO scale, median (Q1, Q3) |  |  |  |
| Pain VAS | 74.0 (51.0, 80.6) | 81.8 (75.7, 89.7) | 77.9 (60.6, 87.8) |
| FACIT-Fatigue | 19.0 (13.0, 33.0) | 7.5 (5.0, 19.5) | 15.0 (7.0, 28.0) |
| DLQI | 19.0 (14.0, 23.0) | 21.0 (17.0, 27.0)^†^ | 19.5 (15.5, 25.0) |
| PSS | 11.0 (9.0, 12.0) | 11.0 (9.0, 13.0) | 11.0 (9.0, 12.0) |
| Body temperature, n (%) |  |  |  |
| ≤38.5°C | 28 (96.6) | 21 (87.5) | 49 (92.4) |
| >38.5°C | 1 (3.4) | 3 (12.5) | 4 (7.5) |
| CRP level, n (%) | ^‡‡^ | ^§§^ |  |
| <3 mg/L | 2 (6.9) | 3 (12.5) | 5 (9.4) |
| ≥3 to <70 mg/L | 18 (62.1) | 10 (41.7) | 28 (52.8) |
| ≥70 mg/L | 7 (24.1) | 8 (33.3) | 15 (23.8) |
| WBC count, n (%) |  | ^¶¶^ |  |
| <10 × 10^9^/L | 14 (48.3) | 6 (25.0) | 20 (37.7) |
| ≥10 to <15 × 10^9^/L | 12 (41.4) | 10 (41.7) | 22 (41.5) |
| ≥15 × 10^9^/L | 3 (10.3) | 4 (16.7) | 7 (13.2) |
| Neutrophil count, n (%)  ≤ULN (7.23 × 10^9^/L)  >ULN (7.23 × 10^9^/L) | 14 (48.3)  15 (51.7) | ^¶¶^  5 (20.8)  15 (62.5) | 19 (35.8)  30 (56.6) |
| Albumin level, n (%)  ≥38 g/L  30 to <38 g/L  <30 g/L | ^‡‡^  22 (75.9)  4 (13.8)  1 (3.4) | ^§§^  16 (66.7)  5 (20.8)  0 | 38 (71.7)  9 (17.0)  1 (1.9) |
| Hospitalized for current GPP flare, n (%) | 11 (37.9) | 14 (58.3) | 25 (47.2) |
| Number of days in hospital for current GPP flare, median (Q1, Q3) | 8.0 (4.0, 10.0)^†††^ | 9.0 (5.0, 13.0)^‡‡‡^ | 8.0 (5.0, 11.0) |
| Number of days in ICU for current GPP flare, median  (Q1, Q3) | 0 (0, 0)^†††^ | 0 (0, 0)^§§§^ | 0 (0, 0) |

^†^Data missing for one participant in the spesolimab group. ^‡^Data missing for one participant in the placebo group. ^§^Data missing for three participants in the spesolimab group. ^¶^DNA sequencing was not performed in three participants in the spesolimab group and one in the placebo group. ^††^DNA sequencing was not performed in three participants in the spesolimab group. ^‡‡^Data missing for one participant in the spesolimab group and one in the placebo group. ^§§^Data missing for two participants in the spesolimab group and one in the placebo group. ^¶¶^Data missing for two participants in the spesolimab group and two in the placebo group. ^†††^n = 11. ^‡‡‡^n = 14. ^§§§^n = 13.

BMI, body mass index; CRP, C-reactive protein; DLQI, Dermatology Quality of Life Index; FACIT-Fatigue, Functional Assessment of Chronic Illness Therapy–Fatigue; JDA-GPPSI, Japanese Dermatological Association Generalized Pustular Psoriasis Severity Index; GPP, generalized pustular psoriasis; GPPASI, Generalized Pustular Psoriasis Area and Severity Index; GPPGA, Generalized Pustular Psoriasis Physician Global Assessment; ICU, intensive care unit; PRO, patient-reported outcome; PSS, psoriasis symptom scale; Q, quartile; SD, standard deviation; ULN, upper limit of normal; VAS, visual analog scale; WBC, white blood cell.
